# Evaluation of an IoT Application-Scoped Access Control Model over a Publish/Subscribe Architecture Based on FIWARE

**DOI:** 10.3390/s20154341

**Published:** 2020-08-04

**Authors:** Alejandro Pozo, Álvaro Alonso, Joaquín Salvachúa

**Affiliations:** Departamento de Ingeniería de Sistemas Telemáticos, Escuela Técnica Superior de Ingenieros de Telecomunicación, Universidad Politécnica de Madrid, 28040 Madrid, Spain; alejandro.pozo@upm.es (A.P.); joaquin.salvachua@upm.es (J.S.)

**Keywords:** IoT, security, identity management, access control, OAuth 2.0, CoAP, publish & subscribe, IAACaaS

## Abstract

The Internet of Things (IoT) brings plenty of opportunities to enhance society’s activities, from improving a factory’s production chain to facilitating people’s household tasks. However, it has also brought new security breaches, compromising privacy and authenticity. IoT devices are vulnerable to being accessed from the Internet; they lack sufficient resources to face cyber-attack threats. Keeping a balance between access control and the devices’ resource consumption has become one of the highest priorities of IoT research. In this paper, we evaluate an access control architecture based on the IAACaaS (IoT application-Scoped Access Control as a Service) model with the aim of protecting IoT devices that communicate using the Publish/Subscribe pattern. IAACaaS is based on the OAuth 2.0 authorization framework, which externalizes the identity and access control infrastructure of applications. In our evaluation, we implement the model using FIWARE Generic Enablers and deploy them for a smart buildings use case with a wireless communication. Then, we compare the performance of two different approaches in the data-sharing between sensors and the Publish/Subscribe broker, using Constrained Application Protocol (CoAP) and Hypertext Transfer Protocol (HTTP) protocols. We conclude that the integration of Publish/Subscribe IoT deployments with IAACaaS adds an extra layer of security and access control without compromising the system’s performance.

## 1. Introduction

For decades, the automotive industry used to design and build cars that could only carry their own load and that of passengers disregarding their protection. We can establish an analogy of this fact relating to the current state of IoT (Internet of Things) devices. IoT is in the process of reaching a balance between hardware resources and security guarantee. In the meantime, some current security technologies could be applied to IoT to enhance the “highway” and preserve the “passengers” safety.

### 1.1. Motivation

IoT involves connecting physical devices (that transfer and receive messages) to the Internet without any human intervention. The large number of messages generated on IoT networks implies a challenge to the design of dynamic, reliable and scalable architectures. Design patterns as Publish/Subscribe are usually well integrated in IoT environments. This pattern brings a lot of benefits for the development of IoT applications to enhance society’s activities but also presents new privacy and security vulnerabilities. IoT botnets, Denial-of-Service or Man-in-the-Middle are typical IoT cyber-attacks that can be mitigated using proper access control mechanisms. IoT application layer protocols should adopt this pattern but also implement access control solutions that should not drastically affect the performance of IoT applications.

Introducing access control mechanisms in IoT devices involves several implications in terms of resource consumption such as central processing unit (CPU), memory or battery lifetime. Certain devices integrate their own authentication and authorization mechanisms; however, they consume an extra amount of these resources and frequently present severe vulnerabilities. Other low-resource devices rely on simple authentication mechanisms based on pre-configured credentials such as IDs, API (Application Programming Interface) keys or username/password. These credentials are sent (generally through a gateway), along with the device information, towards a data system in which are validated. If attackers intercept these requests, they would get the credentials and would be able to perform malicious requests [1]. In this context, IoT can benefit from the introduction of delegated authentication and authorization mechanisms, which do not greatly increase the resource consumption of the device and reduce stealing credentials risks. The OAuth 2.0 authorization framework [2] enables applications to delegate the authorization process with a third party and without sharing passwords of actors (users or machines). OAuth 2.0 defines several flows (grant types) to obtain a credential (token) to be used when accessing the resources of applications. Although the specification is designed for HTTP-based web applications, it could be adapted to be used in other scopes, such as IoT. In particular, the Client Credentials Grant was designed for machine-to-machine communications, while the Device Authorization Grant was designed for smart home devices such as Smart TVs.

The OAuth 2.0 protocol presents a very simple mechanism (scopes) for limiting access to resources. This do not encompass most IoT scenarios, in which there are more complex data structures. Therefore, it is essential to deploy a complementary access control architecture that not only enables checking and validation of tokens but also creation and enforcement of fine-grained policies. In this case, an XACML-based architecture is best positioned to perform this task, while it satisfies the scalability, dynamicity and flexibility requirements of IoT environments [3].

Another key point deals with converging proper IoT communication protocols with access control solutions. IoT protocols, such as MQTT (Message Queuing Telemetry Transport), enables secure many-to-many communications and provides 3 QoS (Quality of Service) levels that allow designing IoT applications taking into account the network reliability and the processing capacity. However, it lacks strong authentication or authorization mechanisms and even limits the integration of other existing ones. In particular, MQTT presents difficulties in integrating delegated access control mechanisms such as OAuth [4,5]. On the other hand, CoAP seems to be one of the most propitious protocols that can easily integrate these mechanisms, as we will analyze in further sections.

Finally, the increased use and diversity of IoT applications is accompanied by an increase in difficulties to handle all data generated by sensors and to control the reactions triggered by the actuators (such as valves, relays or pistons). Due to large number of domains to which it can be applied and the particularities of each of them, defining an access control solution for IoT implies a challenge. Following a well-defined architecture and a standard data model contributes to creating fine-grained access control policies, which then enable the control of requests made by actors over the sensors and the actuators. In this sense, the FIWARE platform (FIWARE: http://fiware.org/) provides a set of tools (known as Generic Enablers) that enables the development of secure and smart solutions on scopes such smart cities, smart industry or smart agrifood. The NGSI (Next Generation Service Interfaces) standard, FIWARE’s flagship, provides management of context information and enables the definition of a common data model for IoT applications.

### 1.2. Main Contributions

In this paper, we evaluate the integration of the IAACaaS (IoT application-Scoped Access Control as a Service) access control model into a Publish/Subscribe architecture to protect communications from IoT devices. The resulting architecture allows the establishment of a secure channel to exchange requests from IoT devices with a Publish/Subscribe broker and to externalize the authentication and authorization processes out of them in order to target a reduction of hardware resource consumption of these.

The IAACaaS model, proposed in previous works [6,7] and implemented through the FIWARE open-source platform, is based on the OAuth 2.0 protocol and relies on an XACML (eXtensible Access Control Markup Language) architecture to enable the administration of fine-grained policies. As explained before, we use this model to secure communications between IoT devices and a Publish/Subscribe broker. However, IoT devices usually present low battery life, protocol diversity and security vulnerabilities. In these IoT scenarios is typical to use gateways, which act as bridges between these devices and the brokers. These gateways ensure system interoperability and reduce the number of IoT devices vulnerabilities. Consequently, they become the most susceptible target to be attacked, thus we focus on securing communications between gateways and brokers using the IAACaaS model. On the other hand, securing devices-to-gateways communications could be ensured by deploying the devices in private networks and using lightweight encryption mechanisms. The definition and appliance of fine-grained authorization policies are out of the scope of this paper, but the work that we present enables the implementation of ABAC (Attribute-Based Access Control) or UCON (USage COntrol) models [8]. To evaluate the IAACaaS model, we propose a performance comparison of gateways running OAuth 2.0 over CoAP (Constrained Application Protocol) and HTTP (Hypertext Transfer Protocol) protocols.

As explained above, the IAACaaS model was already presented by the authors of this paper. This model has been adopted by the FIWARE Identity Manager Generic Enabler official reference. Moreover, it has been implemented in Keyrock (Keyrock GE: https://fiware-idm.readthedocs.io), the reference implementation of such Generic Enabler. This implementation has been widely adopted by a lot of IoT and Industry 4.0 success stories among Europe. However, until now there is not any performance and security evaluation of this model. In this paper, we present a case of study using IAACaaS model Keyrock’s implementation and evaluate its performance in terms of security, reliability, latency and CPU, memory and bandwidth consumption. This evaluation can serve as a reference for developers that aim to deploy FIWARE-based sensor networks and therefore using IAACaaS-based Keyrock Generic Enabler.

We use the FIWARE Orion Context Broker, which is the core component of the FIWARE platform and implements the NGSI standard, along with the FIWARE IAACaaS implementation (based on Identity Manager Keyrock and Wilma PEP-Proxy) to deploy the Publish/Subscribe architecture that we evaluate in this paper. In particular, the Orion Context Broker is one of the nine CEF building blocks (CEF Building Block: https://ec.europa.eu/cefdigital/wiki/display/CEFDIGITAL/Context+Broker), whose aim is to help to implement the most commonly needed digital capabilities among Europe. In addition to this, FIWARE is integrated onto the International Data Spaces (International Data Spaces: https://www.internationaldataspaces.org/) initiative whose purpose is to offer a common framework for IIoT (Industrial IoT). In view of all this, FIWARE is becoming one of the most relevant frameworks for the development of IoT applications.

The document is structured as follows. We review the literature on Section 2. Then, in Section 3 we describe the integration of the access control model with the Publish/Subscribe broker. Section 4 describes the technical details of our evaluation and in Section 5 we present its results. Finally, in Section 6 we make a conclusion and explain some possible future lines of research.

## 2. Related Work

Security, and, consequently access control, is the major concern on IoT field [9]. It does not exist a unique access control solution applicable to the wide range of IoT applications, but ABAC or UCON paradigms are the most appropriated to satisfy the demanded IoT requirements [3]. IoT access control solutions must support and implement these paradigms and must be consistent with the IoT’s own protocols and architectures. Standards as OAuth 2.0 [2] or XACML [10] combined with these paradigms allow creation of a fine-grained access control model over IoT Publish/Subscribe architectures [6,7,8]. Moreover, the International Data Spaces created an IIoT standard [11,12] in which they define a vocabulary and data model—implemented with the NGSI FIWARE standard [13]—that boost and eases the definition of fine-grained access control policies with ABAC or UCON [8,14].

Access control cannot be understood without ensuring confidentiality and integrity on data transmission. A non-secured IoT Publish/Subscribe system faces several threats: malicious publishers flooding the network with bogus data (Denial-of-Service attack), malicious publishers generating fake data or malicious intermediaries conducting a Man-in-the-Middle attack [15,16]. Much of the current literature on access control for IoT Publish/Subscribe systems pays particular attention to alleviate these issues. Pesonen et al. propose [17] and validate [18] a device transmission encryption at the attribute level rather than per event. The authors of [19] design three security levels (for their MQTT-based architecture) that each participating entity applies depending on its resources. The study [19] propose an access control model based on the OASIS (Organization for the Advancement of Structured Information Standards) standard and the RBAC (Role-Based Access Control) paradigm. Rizzardi et al. [20] made more comprehensive research which, in contrast to the end-to-end communication of previous ones, deploys an XACML-based architecture in a gateway to establish secure communication with a MQTT broker. This latter research is closer to a real constrained scenario as long as devices and gateways are in a trusted network. The authors of [17,21] reduce communication overhead in some cases, but their solutions would find many difficulties to adapt to typical IoT dynamic and multi-domain environments [3]. In addition, the availability could be compromised as they do not define mechanisms to customize the periodicity or the amount of data to be published by devices [22].

With respect to IoT applications protocols and particularly CoAP, we found that some research develops a Kerberos-based access control model [23,24]. However, these works mainly focus on the users’ side where there are more resources to deploy strong authentication and authorization mechanisms. In addition, Kerberos has a single-domain character that do not fit with a considerable number of IoT applications.

Regarding OAuth integration on IoT applications, a considerable amount of literature has been published on protocols like HTTP [25,26], MQTT [4,5,27] or CoAP [28,29]. Works reported by [26,27,28] focus on OAuth which unlike OAuth 2.0, uses strong signing algorithms that may affect devices performance. The authors of [4,5] points out that a pure Publish/Subscribe pattern (as MQTT) does not support OAuth 2.0 because it was designed for HTTP. In their solution, they had to modify the authorization protocol to fit it with the flows and specifications of MQTT. Navas et al. [29] uses the lightweight COSE (CBOR Object Signing and Encryption) to protect the OAuth 2.0 tokens along with the data to be transmitted through CoAP. Their approach could hinder the scalability because, and, in contrast to DTLS solutions, the application initiates the handshake with devices so it must previously register all devices’ address. The articles [25,27] shows a superficial scheme of how to integrate the OAuth 2.0 protocol and lacks any implementation and validation.

In any OAuth 2.0 implementation is mandatory to ensure communication confidentiality. The IoT applications protocols adopt different standards [30]; MQTT, AMQP o HTTP, as they mainly rely on TCP, adopt TLS [31] while CoAP, as it mainly relies on UDP, adopts DTLS [32]. However, few CoAPs implementations support DLTS [33] and usually lacks session resumption flow [33] which fairly reduce latency and overhead of communications. Furthermore, the TLS/DTLS handshake to establish secure communications and encrypting data transmission consume several resources that are not supported in most of the constrained devices. Some works aim to solve this issue by using a gateway for delegating the initial handshake [34,35] or for performing a unique handshake and maintain the secured socket open [36]. The latter is feasible if it is deployed on trusted networks or if a lightweight secure encryption is implemented between devices and gateways [17,21].

In terms of performance of IoT application layer protocols, authors of [37] performs a comparison between MQTT y CoAP. In particular, they compare the 3 QoS MQTT levels with the CON (Confirmable) and NON (Non-Confirmable) CoAP request types. They state that MQTT should be the preferable protocol for multicast communications. In terms of reliability, MQTT obtains better results than CoAP, especially when the frequency of sending requests is high. This is because MQTT is mainly based on TCP and has a sophisticated congestion control mechanism. However, when the request frequency is low, this improvement is less noticeable. On the other hand, CoAP presents better results in terms of bandwidth usage and round-trip time.

Finally, optimizing devices’ security is as important as ensuring that the access control model can absorb and process all the requests performed by devices. Cloud computing allows scaling access control deployments so that the overall performance of the system is not affected and compromised [38]. On the other hand, fog computing is used on the network’s edge to process some devices’ requests and reduce communications’ latency, so it could be also used to implement an access control solution [39]. In the literature, we can even find security solutions specifically designed for fulfilling the requirements of cloud-based IoT environments, where security is even more critical due to the distributed nature of the devices and management components [40,41].

Publish/Subscribe-based IoT architectures requires lightweight or delegated security mechanisms that should be in concordance to devices’ limitations and should prevent cyber-attacks. They should integrate access control paradigms as ABAC or UCON, in conjunction with other standards as OAuth 2.0 or XACML, which allows defining fine-grained policies over publishers and subscribers. Authorization standards, as OAuth 2.0, implies robust secured communications so the ideal end-to-end communications (between cloud or applications with devices) are not usually feasible in most devices due to theirs constrained feature and it is needed to delegate the establishment of secure communications with external nodes as gateways. On the other hand, CoAP presents a duality and can adopt the Request/Response pattern or the Publish/Subscribe pattern [30], thus it/is able to integrate the OAuth 2.0 easier than MQTT and, additionally, consumes fewer resources than HTTP as we will demonstrate later. In view of all that has been mentioned so far, one may suppose that CoAP is one of the most appropriated protocols to use and secure IoT applications.

## 3. Architecture and Implementation

In this section, we present an integration of the IAACaaS access control solution [6,7] within an IoT-based architecture based on the Publish/Subscribe paradigm. There are increasingly more voices that claim a “secure by design” methodology when developing IoT applications. In designing an IoT architecture, we should take into account the security considerations from the beginning: securely store credentials, securely communicate or minimize exposed attack surfaces. The proposed architecture complies with these security key designs; it allows devices to securely communicate with data systems and to minimize attacks while ensuring the scalability and dynamicity required on IoT applications. In particular, the uni-directional approach design of IoT communications proposed fairly reduced exposed attack surfaces. Due to this characteristic, the architecture is more appropriate for IoT domains in which devices are usually managed centrally, as smart buildings or smart industry.

This paper focuses on evaluating the model from the devices’ side, but the model is also able to control access by users, applications or other services. In this section, we describe some of the main features of the model but more details about the workflows and the components of the IAACaaS model can be found in the works mentioned above. This model guarantees the authenticity, confidentiality and integrity of the IoT communications from a gateway to a Publish/Subscribe broker.

Essentially, the IAACaaS model aims to offload the computational load of the devices by delegating the authorization process to a third party. The model relies on the OAuth 2.0 framework to enable devices to securely request tokens which are used as a proof of authenticity. When the device uploads or ask for data to a service, it includes the token in the request which is validated and authorized through an XACML architecture, before redirecting the request to the service final endpoint. Another important point in which the model relies on to reduce computational load is that devices must not listen for incoming requests. They are only able to periodically send requests either for providing data (sensor) or to check if they should change their state (actuator). This uni-directional approach design brings some benefits that we discuss in the results Section.

The asynchronous Publish/Subscribe pattern has been broadly adopted to implement IoT applications for its capacity to scale. This pattern decouples message senders (publishers) from receivers (subscribers) participating in the communication using an intermediate broker that stores the generated messages. The publisher only concerns about sending the messages, while the broker is responsible for delivering these messages to the proper subscribers. In this manner, subscribers avoid getting stuck waiting for information. However, security is usually an issue as publishers have no knowledge of who is receiving the messages and subscribers have no knowledge of who is generating them. The IAACaaS model boosts and eases the integration of security mechanisms for Publish/Subscribe-based applications.

The IAACaaS architecture are evaluating was originally designed based on the HTTP protocol. However, for the purpose of this paper, we have developed modifications to integrate CoAP, so that we can do a more complete analysis of the access control solution. CoAP is a lightweight protocol that easily interfaces with HTTP, so it can integrate specific HTTP frameworks, such ass OAuth 2.0 with slight modifications. Additionally, it presents a Request/Response-Publish/Subscribe pattern duality which allows it to adapt to a wide range of scenarios. In our case, we use the Request/Response pattern to request access credentials through OAuth 2.0. We use the Publish/Subscribe pattern to update (publish) data through a secure channel including the previous obtained credentials. Other devices could also securely subscribe to the published data.

**Figure 1:**
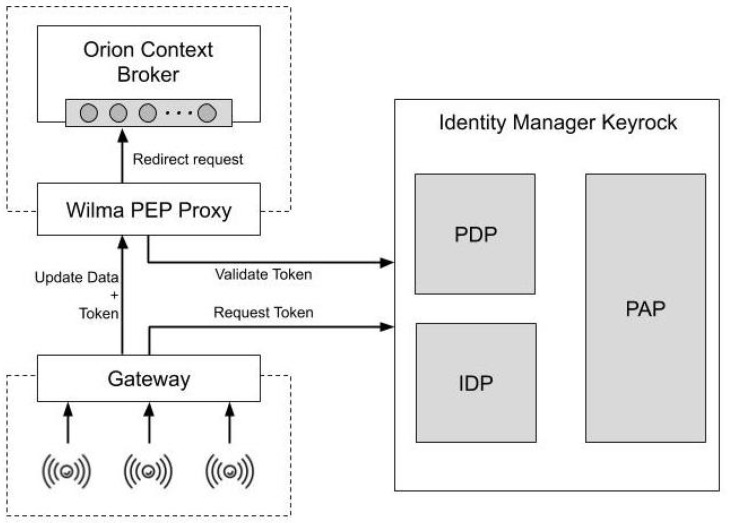
Architecture.

In the architecture we evaluate, both the IAACaaS model and the Publish/Subscribe broker are implemented using the GEs (Generic Enablers) provided by FIWARE, while the gateway is developed using open-source tools. Figure 1 represents the resulting architecture and roughly describes the interactions between the Generic Enablers and the gateway.

### 3.1. Components

We use the following FIWARE GEs to implement the proposed architecture: Identity Manager Keyrock, Wilma PEP (Policy Enforcement Point) Proxy and Orion Context Broker. Keyrock and Wilma have been fully developed and maintained by the authors of this manuscript. Both GEs mainly used the HTTP or HTTPs protocols but, for the purpose of this research, we have modified their source code to support CoAPs (CoAP over DTLS) requests and to integrate OAuth 2.0 over CoAPs.

#### 3.1.1. Orion Context Broker

The Orion Context Broker (Context Broker Orion: https://fiware-orion.readthedocs.io/) is an HTTP Publish/Subscribe implementation—based on the NGSI standard—that enables management of the entire lifecycle of context information including updates, queries, registrations and subscriptions. As explained in Section 1, it has been recognized as a CEF Building Block, which is one step forward on its path towards becoming a global standard for large scale contextual information management. Orion allows defining a model of data (i.e., entity) to which publishers update values to be obtained by subscribers. Orion uses the NoSQL MongoDB database to store these entities and the last value recorded on them. In this architecture, it acts as an intermediary node between the devices (that communicate with it through the gateway) and the applications, so that both of them are able to update or request information from entities.

#### 3.1.2. Identity Manager Keyrock

The Identity Manager Keyrock (IdM Keyrock: https://fiware-idm.readthedocs.io/) is an OAuth 2.0-based identity and access control software, which enables applications and services to delegate authentication and authorization processes. It plays three main roles: IdP (Identity Provider), PAP (Policy Administration Point) and PDP (Policy Decision Point). The two latter enable the creation of access control policies and determine whether or not to authorize a request. For our study, Keyrock also implements the OAuth 2.0 Client Credentials Grant, which enables token creation for machine-to-machine communications without any user interaction.

Keyrock is an HTTP-based server built with Node.js (Node.js: https://nodejs.org/) and relies on a SQL (Structured Query Language) database to provide persistence. Keyrock was modified to support CoAP and DTLS using the *node-coap* and the *node-dtls* libraries, respectively.

#### 3.1.3. Wilma PEP-Proxy

The Wilma PEP Proxy (PEP-Proxy Wilma: https://fiware-pep-proxy.readthedocs.io/) aims to protect services by applying proxy functions based on the OAuth 2.0 protocol and enforcing requests. It can check the identity of the OAuth 2.0 token with Keyrock and to check policies with a PDP. In our case, for the sake of simplicity, Keyrock is also responsible for the PDP functions but FIWARE provides the AuthZforce GE (PDP/PAP AuthZforce: https://authzforce-ce-fiware.readthedocs.io/en/latest/) which also can play this role. It enables a more fine-grained definition of policies based on the XACML language syntax. In our architecture, Wilma control access towards the Orion Context Broker and provides a token cache function that avoids continuously validating tokens with Keyrock.

As well as Keyrock, Wilma is implemented using Node.js and uses the same libraries for CoAP and DTLS support. In our use case, Wilma can act as an HTTP-to-HTTP proxy or as a Cross-Protocol proxy between CoAPs and HTTPs (described in chapter 10 of CoAP’s RFC [42]). Regardless of whether Wilma received CoAPs or HTTPs requests from the gateway, it uses the HTTPs protocol to validate tokens with Keyrock and to redirect requests to Orion. In the case of Orion, it is mandatory as it only supports HTTP/HTTPs, but in the case of Keyrock we decided to use HTTPs to ensure the reliability of tokens validation. However, as future work, we will base the whole architecture in the CoAPs protocol and test it on terms of latency and reliability.

#### 3.1.4. Gateway

The gateway acts as an intermediary node between devices and the Orion Context Broker. It adapts device requests to the NGSI format before sending them to Orion through a secure channel. It requests OAuth 2.0 access tokens from Keyrock, and such tokens are included in the requests sent to Orion. In this paper, we focus on the OAuth 2.0 performance between the gateway and the access control model, so how devices communicate with the gateway is out of the scope of the research. Form the point of view of performance, in many IoT applications a wired installation is infeasible and devices must be wireless, so they should use lightweight secure protocols to connect to the gateway that has minimal impact on energy consumption. However, from the point of view of security and taking into account that the security of a network depends on the security of the most vulnerable node, this issue represents a very important concern. We can found in the literature several studies about the impact of security in wired and wireless sensor networks in which this issue is achieved [43,44,45,46]. On the other hand, the gateway is connected to the Internet and can send requests using the HTTPs or COAPs protocols. If the gateway obtains a response that requires some action, it would notify the smart valve using more lightweight IoT communications based on Zigbee, Bluetooth, etc.

The gateway source code is written in Node.js and relies on *https*, *node-coap* and *node-dtls* libraries to provide either CoAPs and HTTPs requests. In particular, for the CoAPs case, the secured DTLS socket used in the communication remains open similarly to [36].

### 3.2. Flows

We summarize the main flows in the architecture hereafter. A more extended description of the request token and validation token flows can be found in the works mentioned above related to IAACaaS.

- Request token. The gateway uses the OAuth 2.0 Client Credentials Grant to request an access token to Keyrock through a secure channel. To perform that, the gateway is pre-configured with the credentials of an application that must be previously registered in Keyrock. An OAuth client represents that application in the authorization server provided by Keyrock. It owns two principal credentials, an *identifier* and a *secret*. When Keyrock receives a request including these credentials, it checks its validity and generates an access token. Then, the gateway stores the token which will be used in the following flow.
- Update data. When the gateway receives a request from a device, it generates a new request based on the NGSI format. It includes in the body of the request the information received and the access token. The gateway sends the request towards Orion (previously filtered by Wilma). If it receives an unauthorized response, it will request again a new token before performing again the request. The gateway could include information of several devices in the same NGSI request to optimize resources.
- Validate token. Wilma intercepts the request towards Orion and obtain the access token from it. It sends this token to Keyrock to obtain its related information (IdP) and to check its validity (PDP). If it is valid, Wilma stores the token in the cache, along with its expiration time, and redirects the request to Orion. Otherwise it responds to the gateway with an unauthorized response. When Wilma receives again a request with the same token, it follows the same process, but it first checks in the cache if the token is still valid, avoiding the validation with Keyrock.

The presented architecture relies on CoAPs or HTTPs protocols to perform a point-to-point encryption so that confidentiality of communications is preserved. Authentication and authorization processes (based on OAuth 2.0 and the XACML architecture) allow controlling which actor is performing a specific action and provide a comprehensive access control framework.

## 4. Deployment and Use Case

We evaluate our FIWARE-based solution by developing a simple use case for smart buildings. Public and large buildings, such as universities or ministries, usually use central heating systems to regulate the temperature in different areas. These areas are composed of several rooms with several radiators that can be manually managed by opening and closing a valve. There are thermostatic radiator valves, which integrate temperature sensors, that saves considerable amounts of energy [47]. However, they heat the room despite if whether there are people inside or not. Connecting the valves to the network allows control and monitoring of them remotely through an application and would improve the saved-energy results. The application could be reused to control the heating system of all public buildings of a city, so a cloud-based solution is deployed. At this point, we need to introduce an access control model to secure the device side and the user side. We made an experiment focusing on the device side, but the same access control model could be used to control users’ access to the smart valves. If we do not introduce security mechanisms in this application, malicious machines could update fake data to trigger non-desired openings of valves, or to perform a Denial-of-Service attack to the application by sending millions of requests.

In this scenario, we measured the impact on performance by integrating OAuth 2.0 with HTTPs and CoAPs protocol. We define 4 testing scenarios based on request types:

- CoAPs. The gateway sends publish requests to the broker using the CoAPs protocol. Wilma intercepts these requests and generates new HTTPs requests including the payload from the CoAPs ones. The new HTTPs requests are redirected to Orion where they are processed. Finally, the Orion sends a successful response to Wilma that is redirected to the gateway.
- HTTPs. The gateway sends publish requests to the broker using the HTTPs protocol. Then, the flow is the same than in the previous scenario.
- OAuth 2.0 over CoAPs. The gateway retrieves an OAuth 2.0 access token from Keyrock as described in the previous section. Then, it sends publish requests to the broker using the CoAPs protocol including the access token. Wilma intercepts these requests, extracts the access token and validates it. If the access token has expired or if it is not valid, Wilma sends an unauthorized response to the gateway. If the access token is valid, Wilma stores the token in the cache, generates a new HTTPs request including the payload from the CoAPs one and continue the process as described in the CoAPs scenario.
- OAuth 2.0 over HTTPs. The gateway sends publish requests to the broker using the HTTPs protocol. Then, the flow is the same than in the previous scenario.

The two first scenarios (CoAPs and HTTPs) act as a “control group”. They allow us to observe the resource consumption when using those protocols without OAuth 2.0 integration. Comparing them with their homonyms after adding OAuth 2.0 we can isolate the impact of introducing this authorization protocol. On the other hand, OAuth 2.0 was designed for HTTPs so, this in turn will act as a “control group” for the CoAP cases to compare the two protocols.

In the following paragraphs we present the deployment we have designed for the use case on the server-side and the gateway.

### 4.1. Server

We deployed all the server-side components (Orion, Keyrock and Wilma) for the experiment on a cloud provider, so that we can get closer to a real use case. We use an instance of Google Cloud (Google Cloud: https://cloud.google.com) infrastructure hosted in Europe-west1-b (Belgium). Table 1 describes the main specifications of the instance. IoT applications benefit from cloud solutions, as they can dynamically increase the number of instances to support an increment of the number of devices and the number of requests that they generate. In our case, and for the purpose of the experiment, the capabilities of instance that we have deployed are more than enough. Additionally, there is a growing trend to design applications based on microservices and to deploy them using container-based virtualization solutions, such as Docker (Docker: https://www.docker.com) or Kubernetes (Kubernetes: https://kubernetes.io). This approach saves the necessary resources, since it allows scaling up of only those microservices that are overloaded. We use Docker (19.03.7 version) to deploy all the components of the experiment infrastructure and Docker-compose (1.25.4 version) to orchestrate the created containers.

**Figure 2:**
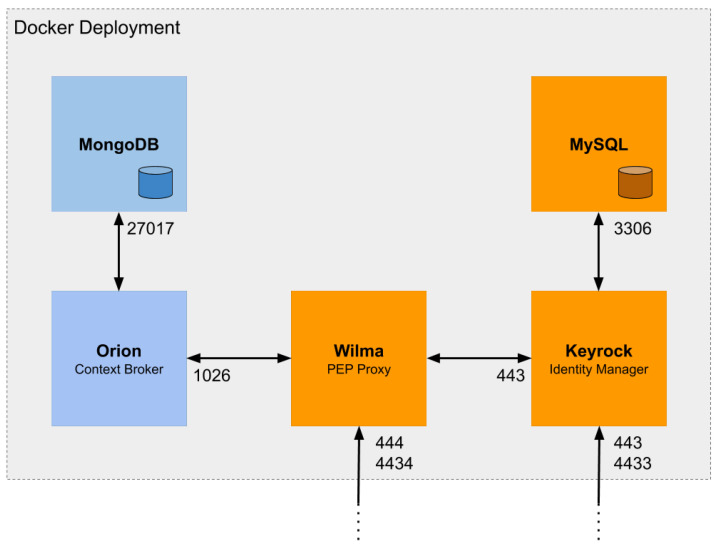
Docker deployment of Generic Enablers.

Figure 2 shows the Docker deployment to install the FIWARE GEs, Orion, Keyrock and Wilma. Below we detail the configurations we have performed for the purpose of the experiment and for the use case presented above.

We have deployed two Docker containers, one for Orion (Orion Docker image: https://hub.docker.com/r/fiware/orion) (2.3.0 docker tag release) and one for Mongo (Mongo Docker Image: https://hub.docker.com/_/mongo) (2.6 docker tag release). We have enabled HTTPs on the Orion container to support secure connections. Regarding the proposed use case, we have created an entity that represents one smart valve in a specific room. This entity periodically updates its data based on requests received from the gateway. We have created that entity by sending an HTTPs request to Orion Context Broker including the information for creating the *temperature, valveRadiator, battery* and *period* fields.

We have deployed two Docker containers, one for Keyrock (Keyrock Docker image: https://hub.docker.com/r/fiware/idm) (7.8.1 docker tag) and one for MySQL (MySQL Docker image: https://hub.docker.com/_/mysql) (5.7 docker tag). We obtained the id and secret credentials of the OAuth client by registering a smart valves application in Keyrock and, then, we included them in the gateway’s code. The size of the OAuth 2.0 credentials is 36 bytes (both for id and secret) while the size of an access token is 40 bytes. These OAuth 2.0 client credentials are usually included on the authorization header (encoded to base 64) in every request for access tokens. However, the CoAP’s headers do not support the size of the credentials, so we enabled Keyrock to obtain these credentials from the body as described in the OAuth 2.0 reference. Therefore, Keyrock can grant tokens either by CoAPs or by HTTPs.

We have deployed a Docker container for Wilma (Wilma Docker image: https://hub.docker.com/r/fiware/pep-proxy) (7.8.1 docker tag). We configured the Keyrock and Orion endpoints and we enabled the token cache functionality. We also took into account the following considerations:

- As we want to check the impact of introducing OAuth 2.0 in the flow, we must compare the same scenario without granting and validating tokens. Wilma can be configured to avoid validating tokens and just redirecting the request to the final endpoint.
- When we deploy the scenarios of CoAPs and HTTPs without OAuth 2.0, we configure Wilma to redirect the incoming requests to the final endpoint without performing any token validation.
- When Wilma validates a token with Keyrock, it obtains the token expiration time. Wilma supports a customizable token cache function that prevents checking every request with Keyrock. On this cache, it stores the token and the expiration among other things. Whenever Wilma receives a token it checks if it is stored in the cache and if it is valid. In case that the token has expired, Wilma responses with an unauthorized code.

Storing great amounts of tokens in the PEP’s cache and IdM’s database can affect the overall performance of the system. As docker-compose enables the creation and destruction of the test scenario in an easy way, we made each test in a clean environment. However, in a real scenario, cache cleaning or database management should be done without stopping the service.

### 4.2. Gateway

We deployed the gateway for the experiment on a Raspberry Pi 3 (Raspberry Pi: https://www.raspberrypi.org/products/raspberry-pi-3-model-b-plus/) and we used its WiFi interface to send requests to the cloud deployment. Table 2 shows the Raspberry capacities. These capacities far exceed the capabilities of commercial gateways, so we limited them to better approximate to a real case. In a production deployment, it should be estimated the number of nodes that the gateway would support to avoid oversizing the hardware. For this purpose, it should be taken into account criteria such as: the communication protocols used by the sensors, the number of requests per second made by each of them or the size of the payload of the requests [48,49]. In the specific case of the deployment we have made and the measures obtained (which will be presented in the next section) regarding CPU, bandwidth and memory, we can estimate that the gateway would support in the order of dozens of IoT devices.

As we stated before, in this paper we focus on the OAuth 2.0 performance between the gateway and the access control. In a production deployment, the gateway would adapt the valves-messages to the NGSI format before publishing them using the HTTPs or COAPs protocols.

As OAuth 2.0 requires secure connections, either for CoAPs and HTTPs we use the RSA algorithm for key exchange and authentication, along with the encryption 128-AES-CBC and SHA. In a real scenario, a more efficient and secure key exchange and encryption algorithm should be used. For instance, it would be more suitable to use elliptic curve Diffie-Hellman [50] for key exchange and stronger hashing algorithm as SHA256 [51].

We also developed a python script (based on the psutil library) to monitor the execution of the gateway’s code. This script periodically measures CPU, memory and bandwidth performance. Once a test has been completed, all the measures are stored into a CSV (Comma-Separated Values) file. On the other hand, we have included in the code of the gateway itself the necessary logic to measure the latency of the requests.

## 5. Results and Analysis

For evaluating the presented scenario, we made an experiment consisting of periodic send requests from the gateway to the deployment made in the cloud. We evaluated the scenario in terms of CPU, bandwidth, memory, latency and reliability and in 4 scenarios explained in the previous section: CoAPs, OAuth 2.0 over CoAPs, HTTPs and OAuth 2.0 over HTTPs. We have also considered the following configuration:

- The duration of each test is 5 min.
- We modify the period of the requests for each of the scenarios. The period values are 0.1, 0.2, 0.5, 1 and 2 s.
- The size of the update requests payload is 232 bytes for OAuth 2.0 cases and 174 bytes for non-OAuth 2.0 cases. The payload of the access token request to Keyrock is 143 bytes while the response is 60 bytes.
- Wilma responses to the gateway with a 204 HTTP code (2.04 in CoAPs cases) if the entity has been successfully modified in the context broker. Otherwise, Wilma responses with a 401 HTTP code (4.01 in HTTPs cases) if the access token is no longer valid.
- The time a token could be stored in the Wilma token cache is higher than the expiration time. Therefore, when Wilma receives a request with a token and it checks in the cache that this token exists and has expired, it responds with an unauthorized code to the gateway.
- For the OAuth 2.0 cases, we set the token expiration time in 10 s. The gateway requests a new access token to Keyrock each time the PEP-Proxy responds with an unauthorized code. As each experiment lasts 5 min, it has been necessary to set such a low expiration time to appreciate OAuth’s impact. However, in a real scenario, in which the gateway would have a continuous operation over time, the token expiration time should be much greater, which would allow enhancement of the IoT application’s performance. Establishing a higher token expiration time does not pose a risk to token leakage and improves system performance. On the other hand, refresh tokens or non-expiring access tokens could be used. Refresh tokens (with a longer expiration time) can be used to obtain a new access token without performing again the authentication. Non-expiring access tokens allow drastic reduction of the number of authentication requests but it is necessary to provide a revoke mechanism [52].

For getting the experimental data, at the same time we execute these scenarios in the gateway, we also run the python monitoring script described in the previous section. This script periodically (every 0.2 s) takes the values of CPU and Memory along with the bandwidth sent and received. When the execution is completed, the values are stored in a CSV file. This file is cleaned and processed to perform an operation over the data obtained. On the other hand, we store in an array the latency of each requests as well as the response codes (HTTP or CoAP) of each them. A CSV file is generated again for being then cleaned and processed to analyze the latency and the reliability of each scenario.

To determine and compare the performance of each of the 4 scenarios, it is necessary to know the latent performance of the Raspberry itself. We measure the performance of the Raspberry with the WiFi interface activated and when executing the python monitoring script. During a period of 5 min, the Raspberry consumes an average of 3.84% of CPU and 21.64% of Memory while the bandwidth consumption is negligible.

### 5.1. Bandwidth

**Figure 3:**
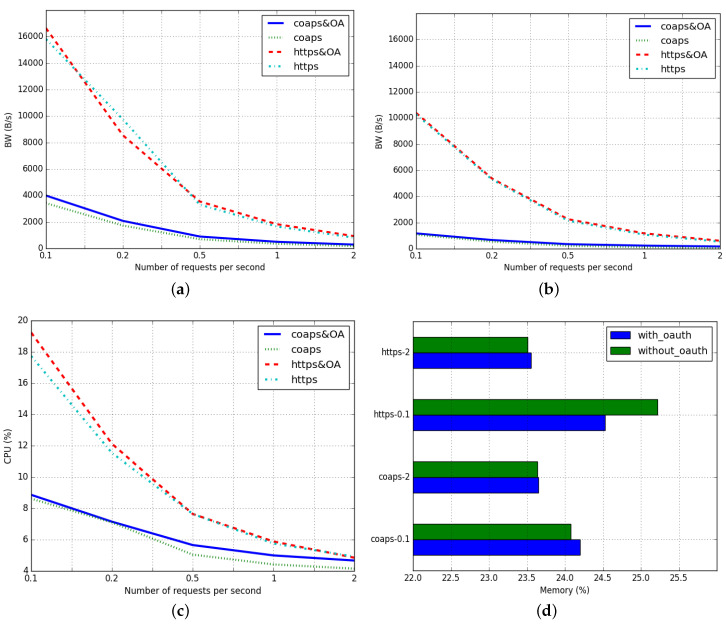
Bandwith, CPU and Memory performance. (**a**) Mean of BW sent; (**b**) Mean of BW recv; (**c**) Mean of CPU; (**d**) Mean of Memory.

We first investigated the impact on bandwidth consumption comparing a scenario in which the gateway uses OAuth 2.0 with a scenario in which it does not. We measured the number of bytes sent and received per second on the WiFi interface of the Raspberry. Figure 3a,b show the evolution of the bandwidth’s means for each periodicity and for each request type. As expected, CoAPs consumes less bandwidth than HTTPs especially when the periodicity increases, but the incidence of OAuth 2.0 on the percentage of bandwidth is slightly higher in CoAPs. For instance, with a period of 0.1, OAuth 2.0 increases by 16% the bandwidth in CoAPs case, while in HTTPs case it increases by 6%. This occurs because the overhead of a CoAPs request is smaller than the HTTPs one, so it is more notorious to introduce an extra load on it. We can see that the OAuth 2.0 provides an extra access control layer without supposing a considerable decrease on bandwidth performance.

### 5.2. CPU

Regarding the impact of using of OAuth 2.0 in terms of CPU, we measured the overall performance of the Raspberry, which includes the proper performance of the monitoring script. Figure 3c shows the evolution of CPU performance’s means for each periodicity and for each request type. As the previous case, CoAPs consumes fewer CPU resources than HTTPs, especially when the periodicity increases. However, we can observe a different behavior when including OAuth 2.0 on each protocol. In HTTPs cases, OAuth 2.0 supposes an increment on CPU performance as the request periodicity decreases while in CoAPs cases, it causes an increment as the request periodicity decreases. We deduce that using a stateless approach (as the HTTPs ones) suits better when the request density is low while remaining the socket open (as the CoAPs ones) suits better when the request density is high. If the gateway dynamically changes the approach based on the density of requests, the CPU performance could be greatly improved including the one produced by OAuth 2.0 itself.

### 5.3. Memory

In terms of memory, we measured again the overall performance of the Raspberry, which includes the proper performance of the monitoring script. The means of the memory values obtained have a low variability, while some of them have different values than expected. This is due to external factors of the execution that have affected slightly some of the measurements. To better appreciate the impact on memory, we decided to use a bar graph—instead of a linear graph—to show the extreme values of the density of requests. Figure 3d shows the particular cases for the 0.1 and 2 s periodicity and we observe a similar behavior as the CPU performance. The HTTPs approach improves memory usage when the density of request is low while the CoAPs one improves it when the density of request is high. We observe that the HTTPs-0.1 case presents values contrary to those expected, being that the non-OAuth 2.0 case uses more memory than the OAuth 2.0 case. This is due to the variability issue that we have commented before. Regarding the rest of the cases shown, we can state that OAuth 2.0 slightly increases memory consumption, but it is bearable. In future works, we will study the OAuth 2.0 memory impact when using a larger token size and different encryption algorithms. These actions may affect the performance of the gateway, but they could improve the integrity of the communications as well.

### 5.4. Latency

**Figure 4:**
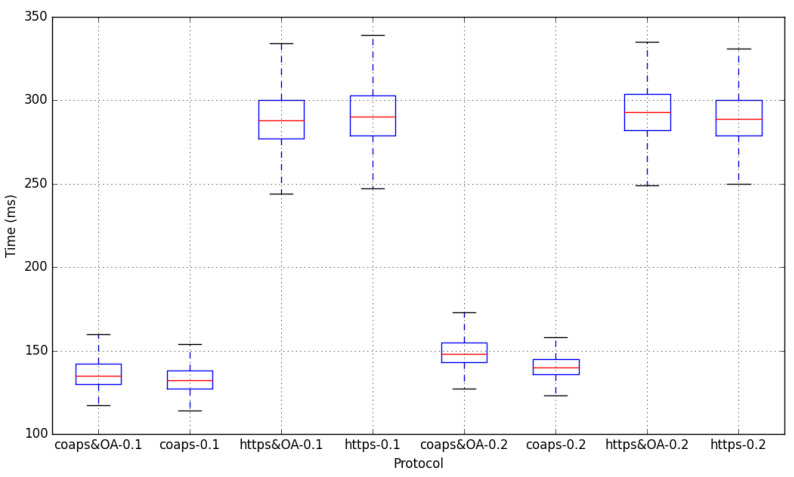
Latency for 0.1 and 0.2 s period.

We also measured the end-to-end latency introduced by the OAuth 2.0 protocol. We estimated latency as the time lapse between the gateway starts configuring the update request and the response obtained from Wilma. For the OAuth 2.0 cases, the elapsed time for requesting a token to Keyrock is added to the subsequent Wilma update request. Figure 4 shows a comparison between the use of 0.1 and 0.2 s for the period and for each type of request. We can see that there is an expected difference of ∼150 ms between CoAPs and HTTPs. The OAuth 2.0 incidence for the HTTPs cases is almost negligible, while in the CoAPs is slightly higher. Again, as in the case of the bandwidth, the lower overhead of CoAPs explains this behavior. We can also see that the 0.2-period case of CoAPs has a higher value (with a ∼25 ms) than the 0.1-period one. This can be also explained with the token expiration issue. Wilma token cache allows considerable reduction of the latency of introducing OAuth 2.0. For instance, when the gateway sends a CoAPs request including a new token (i.e., when Wilma needs to validate the token with Keyrock), it usually takes ∼700–800 ms, while including an old token (stored in Wilma token cache) takes around ∼120–150 ms. We can conclude that the cache mechanism reduces latency of the access control solution.

### 5.5. Reliability

We have made a reliability analysis of the answered requests in the OAuth 2.0 scenarios. The results are shown in Table 3. As we can see, all update requests have been successfully responded but, for the HTTPs case using 0.1 s and 0.2 s periods, we note that the gateway has received more 401 codes than the expected (since the token expires after 10 s and the duration of the test is 5 min, it should be at most 30 responses with 401 code). We relate this behavior with the higher latency of HTTPs. When using a low frequency, during the time frame in which the gateway receives the first unauthorized response it continues sending requests. For the CoAPs case, this behavior is beginning to be observed at 0.1 s periods. In this case, a solution could be that the gateway stores the token expiration time when retrieving a token from Keyrock and checks this time before performing any request. In addition, it must integrate synchronization mechanisms such as the NTP (Network Time Protocol) [53] to get the same time as the cloud deployment. We do point out that in the future we will do more intensive tests undo different conditions: by increasing number of requests, by increasing congestion of network, by limiting the bandwidth, etc.

These measures have been taken without any network congestion. The authors of [37] emulate a congestion scenario using NetEM (Network EMulation) in order to determine packet loss when using CoAP and MQTT. They configure this software to randomly drop 20% of incoming packages. They discovered that using CoAP under these circumstances supposed a packet loss of 35% when the request frequency was 5 s. This is mainly due to UDP reliability. However, when the frequency of requests increased, the packet loss was significantly reduced. In view of this and in a real scenario, the gateway we have deployed would have to decrease the requests frequency to avoid a possible package loss.

### 5.6. Discussion

In this subsection, we summarize some of the disclosures of the experiment regarding OAuth 2.0. Even though the gateway would support HTTPs and as OAuth 2.0 could run over CoAPs, using the second one would allow the gateway to support a higher amount of smart radiator valves. Likewise, the gateway should also change dynamically between a stateless CoAPs approach and remaining the UDP socket opened, based on the density of the number of requests per second. This functionality would not only enhance the OAuth 2.0 performance but also that of the whole system. Another important point is about token time expiration, which should be carefully configured to ensure the performance and the integrity of the application. The gateway may also handle this expiration time to foresee when it should request a new access token, but this implies it must integrate a synchronization protocol. Related to the two previous points, the Wilma token cache plays an important role as it avoids an unnecessary repetitive validation of tokens when the request’s parameters and the token are the same. Considering the above reasoning we can conclude that OAuth 2.0:

- Allows secure and easy identification of the specific entity performing an specific request.
- Do not require considerable additional hardware capacity to be executed on IoT environments.
- It boosts the integration of comprehensive access controls paradigms as ABAC or UCON.
- Do not interfere on the definition of a data model which is relevant to the implementation of the mentioned paradigms.

Regarding this last point, using a data model to describe the attributes of the smart radiator valve (NGSI), allows the creation of a digital twin stored in the cloud. A digital twin allows easy interface with the IoT device and to monitor the state of the IoT device. In our experiment, the smart radiator valve sends publish requests through the gateway to update its digital twin, but it can also send requests to check if its digital twin has changed—due to users or algorithms actions—the valve opening status and perform the action in question. Likewise, the smart radiator valve can update information about the remaining battery life to its digital twin allowing for predictive maintenance. On the other hand, the access control can be extended to enforce and validate policies with the aim of limiting access to the devices attributes. Creating a digital twin of the device eases the definition and application of fine-grained policies and, as the device is not awarding for requests, the risk of attacks such as DoS is reduced. In addition, the digital twin must contemplate configuration attributes of the device that can be changed, such as the periodicity or even the amount of data to be sent, so that users (or even applications in an automatic way) are able to manage the device. The access control policies should contemplate both the device’s data and the monitoring and configuration of the device.

The present study makes several noteworthy contributions to access control on IoT. This combination of results provides some support for securing IoT applications. As we commented in the related work, it does not exist a unique access control solution for IoT applications. The conclusions drawn from the evaluation of the scenarios can be very useful on domains such as smart building, smart home or smart industry. However, more research on latency or privacy needs to be taken to be able to deploy the architecture presented in fields such as Smart Vehicles or Smart Health.

## 6. Conclusions and Future Work

The present study was designed to evaluate the effect of integrating the IAACaaS access control model [6,7] on an IoT Publish/Subscribe scenario from the devices’ side. Devices or gateways usually have more resources limitations than the user side or the cloud, so in this paper, we focus on evaluating that is feasible to integrate the OAuth 2.0 protocol (the basis for the IAACaaS model) on IoT applications without requiring an extra of system capabilities. Although the present study is based on a simple use case, the findings suggest that the OAuth 2.0 protocol adds an extra layer of access control without compromising the performance of the system by itself. It is the establishment of secure connections that most limits performance. As we stated in the Related Work Section, any access control solution (as OAuth 2.0) must support secure communications, so further investigation and experimentation into encryption or key exchange of devices and gateways is strongly recommended. It might be possible to use a different approach as OSCORE (Object Security for Constrained RESTful Environments) [54] which allows establishment of end-to-end secure communications. This standard could avoid the need for introducing a gateway as it could considerably reduce resources consumption.

On the other hand, the study did not evaluate the creation and enforcement of policies so a natural progression of this work is to analyze the impact of introducing ABAC or even UCON fine-grained policies definition and evaluate again the performance of the whole system. The studied use case could be extended to create ABAC policies through the access control model described in [6,7]. Since IoT is growing exponentially, further research might investigate the scalability of the policies compliance in a more complex scenario. Another research line could be introducing MAC tokens [55] (instead of Bearer tokens) with the aim of avoiding the handshake proper of DTLS or HTTPs communications. Finally, comparing the performance of an MQTT-based implementation with the experiments performed in this research would be of interest for with a more complete evaluation of the scenario.

## Figures and Tables

**Table 1:** Cloud instance specifications.

**CPU**	4 virtual CPU
**Memory**	15 GB
**Disk**	30 GB SSD
**Operating System**	Ubuntu Bionic 18.04

**Table 2:** Raspberry specifications.

**CPU**	1 virtual CPU
**Frequency**	600 MHz
**Memory**	407 MB
**Disk**	16 GB
**Operating System**	Raspbian Buster Lite 4.19
**Wi-Fi**	2.4 GHz 802.11 n WPA2 PSK

**Table 3:** Request comparison.

Name	Total-Updates	Status-204	Status-401
**coaps-OA-0.1 s**	2785	2750	33
**coaps-OA-0.2 s**	1412	1382	30
**coaps-OA-0.5 s**	572	545	27
**coaps-OA-1 s**	287	261	26
**coaps-OA-2 s**	144	120	24
**https-OA-0.1 s**	2791	2699	87
**https-OA-0.2 s**	1423	1364	58
**https-OA-0.5 s**	577	547	29
**https-OA-1 s**	284	258	26
**https-OA-2 s**	146	122	24
